# The Effectiveness of Autologous Platelet Rich Plasma Application in the Wound Bed Prior to Resurfacing with Split Thickness Skin Graft vs. Conventional Mechanical Fixation Using Sutures and Staples

**DOI:** 10.29252/wjps.8.2.185

**Published:** 2019-05

**Authors:** Subha Dhua, TR Suhas, BG Tilak

**Affiliations:** Vydehi Institute of Medical Sciences and Research Centre, Bengaluru, India

**Keywords:** Platelet rich plasma, Autologous, Wound, Split thickness skin graft, Graft

## Abstract

**BACKGROUND:**

Autologous platelet rich plasma (PRP) has significant benefits facilitating improved graft take on wound beds due to hemostasis, adhesive and healing properties. This study aimed at effective use of PRP in wound beds on graft take irrespective of etiology as compared to conventional methods of mechanical fixation using sutures and staples.

**METHODS:**

Forty cases including 20 in control and 20 in PRP groups admitted to the Department of Plastic Surgery at Vydehi Institute of Medical Sciences and Research Centre, Bangalore were enrolled between October 2015 and September 2017. Freshly prepared autologous PRP was applied on wound beds in the treated group, while conventional mechanical fixation methods like staples and sutures were used in the control group for the fixation of the skin grafts.

**RESULTS:**

Most significant result was the instant graft take to the wound bed irrespective of the etiology besides hemostasis and healing properties in the PRP treated group which resulted in considerable reduction of surgeon’s time required for the removal of sutures and staples at the final stages. Also, only 10% with graft edema were noted in the PRP treated patients as compared to 68% in the control group. The inner dressings and skin graft were dry in the PRP group and the post-operative etching, weeping and pain at the graft site reduced.

**CONCLUSION:**

The cosmetic appearance of this scar was better in the PRP group besides post-operative edema and graft loss. The study recommends use of PRP at the recipient site of split thickness skin graft.

## INTRODUCTION

Split thickness skin graft is generally done for soft skin coverage in view of its broad application for use due to ease of harvest. The healing process is undertaken through three stages of anchorage,^1^ inosculation,^2^ and maturation.^3^ During wound healing, platelets are activated by contact with collagen. Platelets secrete stored intercellular mediators and cytokines from the cytoplasmic pool and release their α-granule content after aggregation. Also, cell proliferation, angiogenesis and cell migration are stimulated resulting in tissue regeneration. The anti-inflammatory and angiogenetic effects related to the other properties of platelets are well known.^1^^-^^3^

The application of autologous platelet rich plasma (PRP) to the split thickness skin graft sites is considered and theorized to provide immediate skin graft anchorage, as well as inosculation of the split thickness skin graft (SSG) with nutrient rich blood media. This study was performed to report the time to >90% primary healing of SSGs augmented with application of PRP in a high-risk patient population (The mean time to ≥90% SSG recipient site healing was 16±4.2 days as determined by retrospective chart review and digital photograph analysis.^4^ The addition of PRP to SSG recipient sites seems to enhance primary healing and reduce healing time, likely as a result of shearing force reduction and enhancement of the wound environment with growth factors.^5^^-^^7^

Autologous PRP helps to achieve stable hemostasis as it mimics the final steps of coagulation cascade. It brings about instant adhesion of graft to bed preventing any collection under the graft or undue shear.^8^^-^^12^ Chronic wounds may lack growth factors due to decreased production and release, trapping excess degradation or a combination of these mechanisms thus delaying would healing which is overcome by PRP. This study aims at comparing two groups of patients one with and the other without topical application of autologous PRP on the wound bed prior to resurfacing with split thickness skin graft on to assess (i) parameters in both groups i.e. hematoma, discharge from graft site with significant graft loss, (ii) graft edema, (iii) frequency of dressings, (iv) duration of stay in plastic surgery unit and (v) cost effectiveness. In the PRP group, autologous PRP was topically applied on wound beds for graft anchorage, whereas in the control group, the graft was applied and fixed using conventional methods of suturing of staples. 

## MATERIALS AND METHODS

Randomized control study in 40 patients during 24 months from October 2015 to September 2017 was carried for obtaining the necessary ethical clearances. Also, written informed consent was obtained from all the patients included in the study and they were divided into two distinct groups of 20 each and the inclusion criteria were (i) Patients with acute and chronic traumatic wounds, (ii) Infective wounds, (iii) Post-burn wounds, (iv) Wounds following release of post-burn and post traumatic scar contractures, and (v) Co-morbidities like diabetes and hypertension and those on aspirin analogues. Patients who were positive for HIV, HbsAg and HCV and those with coagulation disorders and malignancy were excluded. A randomized procedure was adopted for the 40 patients and was divided into two equal groups of 20 each.

In order to prepare the PRP, aseptic procedures were adopted for drawing the blood preferably from femoral vein alternatively using two 10 ml syringes containing anticoagulant before transferring to 10 ml vacutainers containing 1 ml citrate phosphate, distros-adenine (CPD-A) anticoagulant (freshly obtained from blood bank). PRP was prepared by a double centrifugation process. Whole blood was withdrawn from the patient in 5 ml plain vacutainer tubes. Four milliliter of 3.2% ACD-A anticoagulant was added per vacutainer. The PRP was extracted by a double centrifugation process using a REMI R-303 Table Top Centrifuge. The first ‘hard’ spin was given at 3000 RPM for 20 minutes. After the first spin, the whole blood was separated into 2 layers. The RBCs were collected at the bottom and the plasma buffy coat layer present above was aspirated using a wide bore needle.

**Fig. 1:**
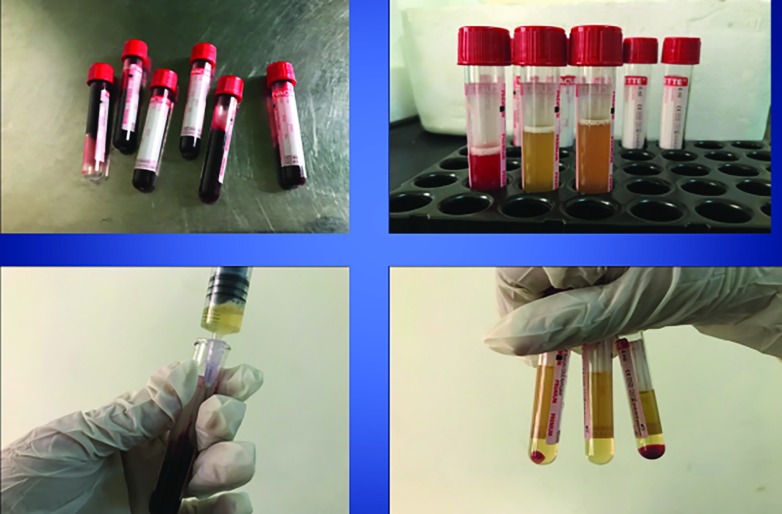
Preparation of platelet rich plasma for application

The transferred plasma was given a second ‘soft’ spin at 1000 RPM for 10 mins. Following this centrifugation, the test tubes showed a collection of platelet pellets with a few RBC at bottom of the tubes with the lower one-third containing platelet rich plasma and the upper two-third containing platelet poor plasma (PPP). The superficial two-third PPP was discarded and the PRP is extracted into a plain test tube. The remaining contents of the tube were discarded 0.05 mL/1 mL calcium chloride was added to the test tube containing the PRP which acts as a clot activator. The PRP was prepared just before the patient was being put on the operating table, since the shelf life of freshly prepared autologous PRP is approximately 2 hours (Figure 1). 

**Fig. 2:**
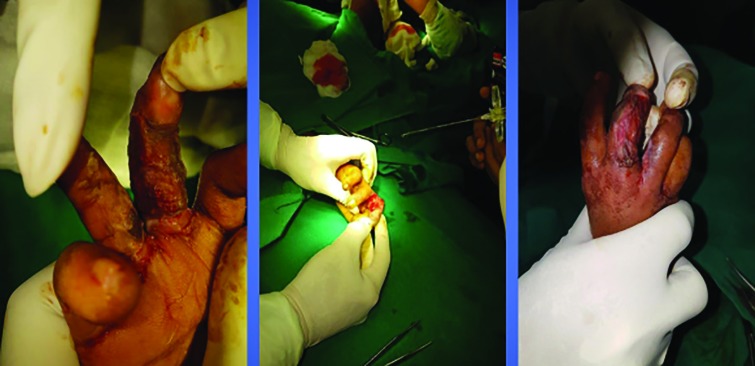
Debridement with simple skin grafting to raw area of middle and ring fingers of right hand with PRP

After the wound beds were prepared, topical application of the harvested PRP was done and the SSGs were placed over the recipient site. The graft appeared to relatively adhere itself to the ulcer bed within a span of 3-5 minutes. This was confirmed by moving the graft with a finger (Figure 2). Once the grafts were secured in place, non-adhesive mesh topped with cotton wool and secured with dressings. Dressings were secured with splints in case grafts were placed over joints. PRP was prepared, while patient was prepped and draped.

**Fig. 3:**
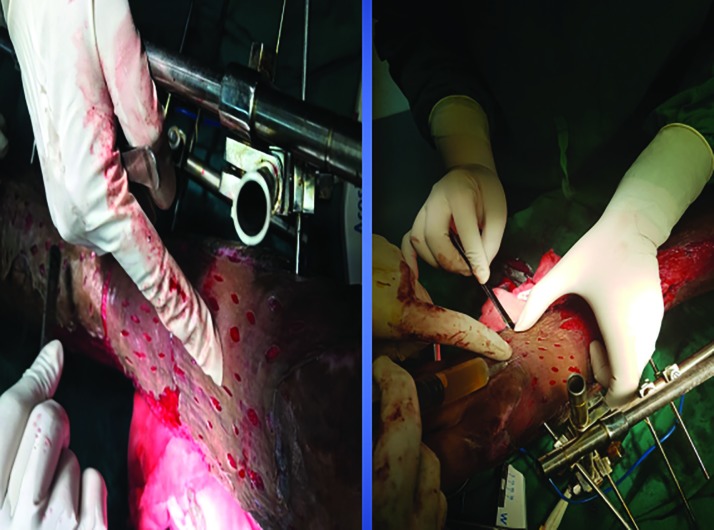
Skin graft being anchored using the PRP under skin graft

In order to compare the effectiveness of PRP with that of the suturing for fixing the SSG, the raw area under the neck and the right forearm were prepared for the placement of SSG harvested from the thigh with which the neck was covered with the SSG and fixed with sutures, while in the lower part of the neck, the SSG was placed after the PRP application for fixation. This procedure helped in the comparison of the effectiveness of PRP in the fixation of the graft with that of mechanical fixation using suturing (Figure 3).

**Fig. 4:**
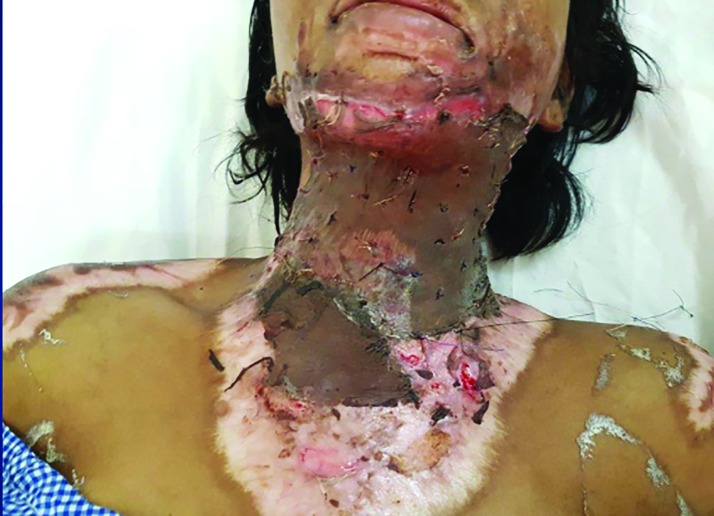
After removing contracture SSG anchored using suturing in the upper part while PRP was used in the lower part for comparison

**Fig. 5:**
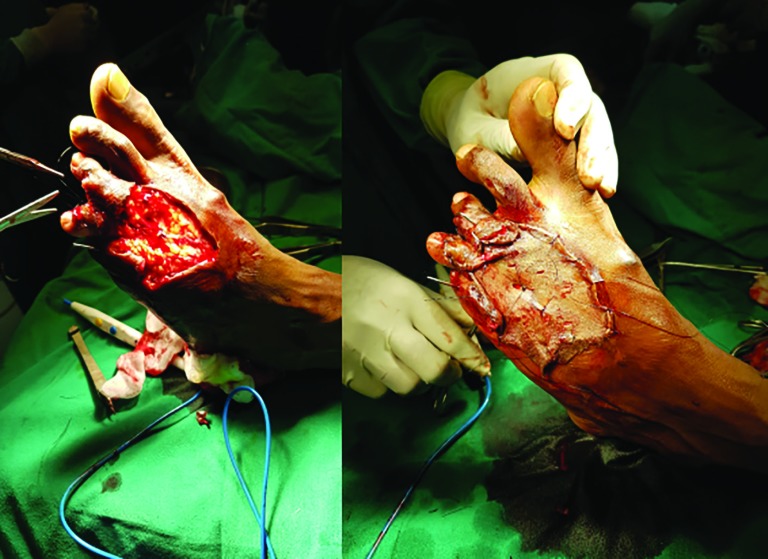
Use of stapling for the fixation of skin graft

**Fig. 6:**
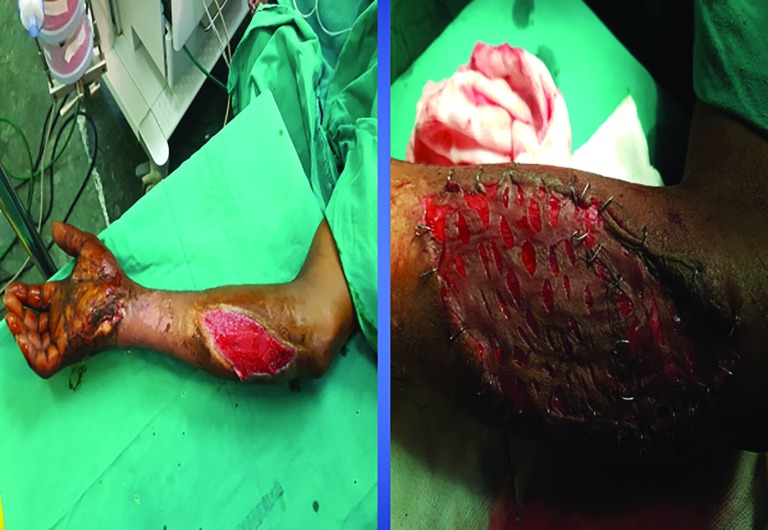
Mechanical fixation of SSG using staples

Regarding the control group, the wounds were surgically debrided, hemostasis was secured and lavage was done for both the selected groups. In the PRP group, application was done topically on the wounds beds through the cannula from the syringe and instant anchorage of skin graft to wound bed was confirmed by moving the graft on the bed with the finger, which was performed by the assistants who were blind to the study. In the control group, sutures or staplers were used to secure the graft to the wound margins and bed (Figure 4-6). In the case of the patients with control group, stitches or staples were used as required to secure the SSG to the wound bed and the margin. Non-adhesive mesh topped with betadine soaked cotton wool was used to secure with compression or tie over bolus dressings as required.

As per the standard practice, the inspection of the graft was carried out during the early post-operative period taking note of the wetness of outer dressing, odor and pain in both the groups. The identified parameters like hematomas and discharge from the graft site, the frequency of discharge from graft site with significant graft loss, graft edema, the frequency of dressings and duration of stay in Plastic Surgery Unit were noted. The number of days of stay in the hospital was specifically noted for both the groups particularly to determine the advantages of using PRP towards the hospital stay, if any, and tabulated. In our study, the patients were followed up for a period of 3 months from the date of discharge to assess any scar hypertrophy in the early post-operative period. 

In order to undertake statistical analysis of data, SPSS software (Version 21, Chicago, IL, USA), in each of the groups were used. The number of patients indicating instant adhesion advantage, graft edema, discharge from graft site, hematoma with significant graft loss and scar hypertrophy were calculated in each group of patients. Hematoma formation, discharge from graft site formation, graft edema, and graft loss between the groups were matched using the Chi square test. Frequency of dressing and duration of stay between the groups was matched using the sample t test. For comparison, computations were done between control and PRP group with regard to day of first graft inspection, frequency of post-operative dressings and stay in plastic surgery unit. Difference in proportion between two groups was tested through Chi-square test. P≤0.05 was considered for statistical significance.

## RESULTS

**Fig. 7:**
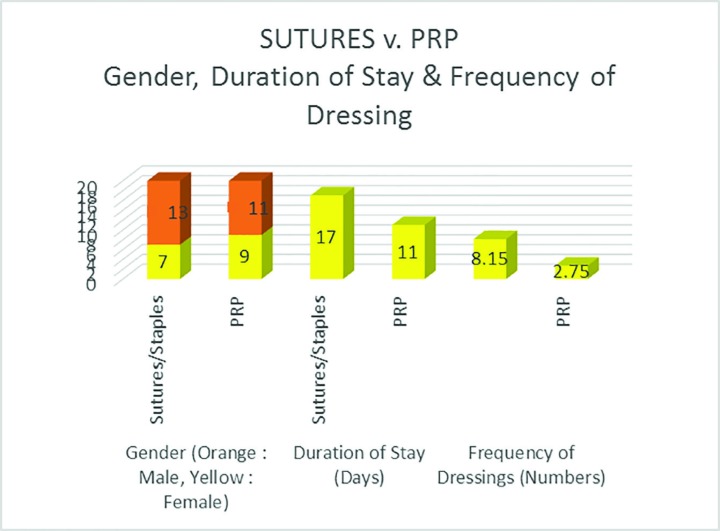
Sutures versus PRP regarding gender, duration of stay and frequency of dressing

In Table 1, type of injury of the patients of the two groups, one with sutures and the staples and the other with the PRP are presented. The gender distribution was presented in Figure 7 and there were altogether 16 females and 24 males out of which in seven patients showed switchers and staples were used in seven females and 13 males, whereas PRP was used in nine females and 11 males. The result of examinations are presented in Figure 7 and it was noted that while there were no ulcer in the PRP group in facia and the muscle, the granulation was 56.25% in cases of the PRP group as compared to 42.75% in the control group.

**Table 1:** Type of injury of the patients of the two groups

**Sutures/Staples**		**PRP**	
**Type of injury**	**No.**	**Type of injury**	**No.**
Degloving injury lower limp	2	Avulsion injury lower limp	1
Diabetic foot ulcer foot	1	Cellulitis right thigh	1
Dupuytrens contracture hand	1	Degloving injury foot	1
Lymphangioma circumscripta Abd	1	Diabetic foot ulcer	1
Naive over neck	1	PBC cubital fossa	1
Non healing ulcer ankle joint	1	PBC let elbow	1
Non healing ulcer heel	1	PBC neck	3
PBC axilla	2	PBC popliteal fossa	1
PBC cubital fossa	1	PBC right arm	1
PBC popliteal fossa	1	Post traumatic raw area thigh	1
Post electrical burn raw area scalp	1	Post traumatic scar forearm	1
Post insect bite raw area scrotum	1	RTA – avulsion right foot	1
Post traumatic constracture foot	1	RTA degloving injury lower limp	2
Post traumatic raw area thigh	1	RTA foot	1
Pressure sore sacrum	1	Traumatic raw area forearm	1
RTA degoving injury thigh	1	Traumatic ulcer lower limb	1
RTA degloving ijury lower limb	2	Traumatic ulcer palm	1
Total	20	Total	20

**Fig. 8:**
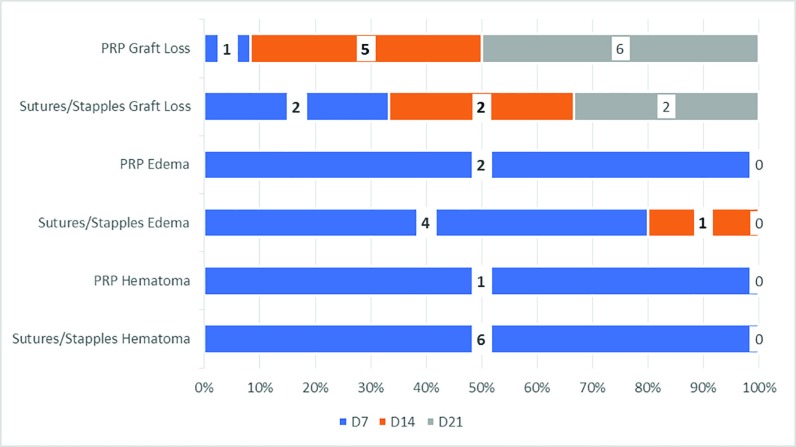
Graft loss in PRP, sutures/sta [especially edema in PRP and sutures and staples and hematoma in PRP and sutures and staples

As seen in Figure 7, the gender in the two groups are comparable (*p*<0.001) with the average age of 38.4 years in the control group and 32.5 years in the PRP group (*p*<0.001). Most interesting part was the duration of stay in which the PRP group had an average duration of stay of 15.25 days as compared to 17.3 days by the control group leading to savings both for the patients and for the hospital in terms of expenditure and satisfaction of the patients. The frequency of change of dressing was 2.75 in comparison to 8.15 with the inner dressings being observed to be significant dry in the PRP group (Figure 8). 

**Fig. 9:**
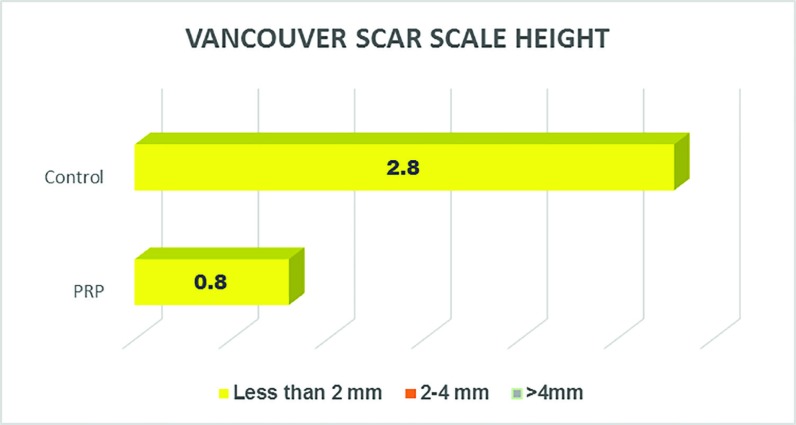
Vancouver sar scale height between two groups

**Fig. 10:**
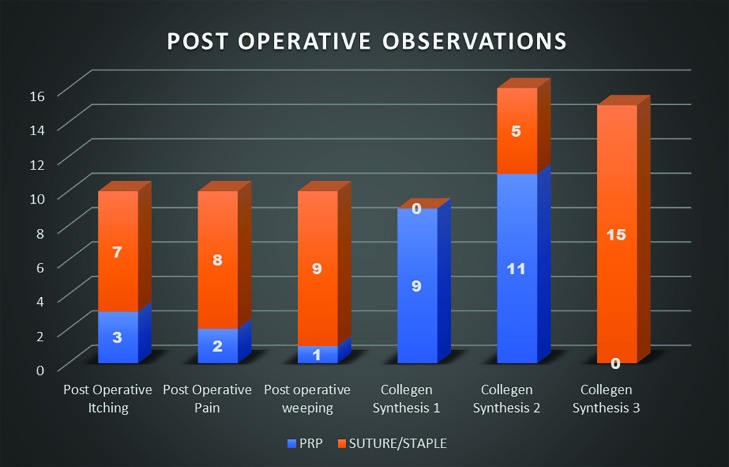
Post-operative observation of the two groups

**Fig. 11:**
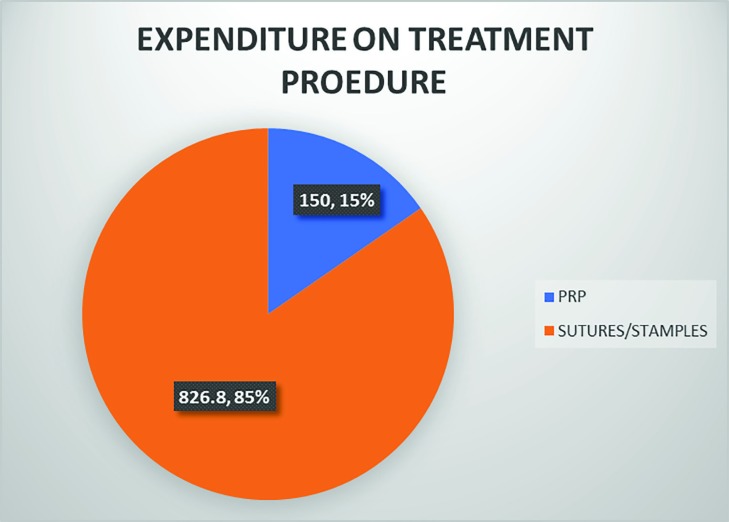
The expenditure on treatment procedure between two groups

The post operative hematoma at the donor site reduced post operative morbidity in the PRP group (*p*<0.05). The edema was much reduced in the PRP group, as compared to the control group (*p*<0.05). With regard to the post operative graft loss, the PRP group significantly reduced the number of losses and the post operating morbidity (Figure 9). The Vancouver Scar Scale height score was <2 mm in the PRP group in all cases as compared to the control group (Figure 10). The post operative itching, pain and weeping at the graft site were considerably less in the PRP group than the control group (*p*<0.05). The figure 11 also shows that the collagen synthesis in the PRP group was significantly more (*p*<0.0001). The total expenditure for the PRP group was around 15%, while that of the control group was 85% (Figure 11).

## DISCUSSION

The entire procedure was aimed at application of platelet rich plasma in surgery prior to resurfacing with skin graft for facilitating instant and stable adhesion of the SSG to the wound bed without any mechanical fixation. The PRP normally has a platelet concentration of about 10,00,000 platelets/µl.^13^ and more than 800 proteins and bioactive factors.^11^ The results clearly showed considerable benefits including hemostasis, speeding up of operating time, frequency of postoperative dressings and the time of the surgery. In our study the preferred anti-coagulant used was CPD-A, because of the fact that the citrate binds calcium to create anti coagulation.^13^

In order to increase platelet activation and the coagulation system in our studies, the blood was drawn immediately prior to the surgery. The supernatant plasma as well as the buffy coat containing high platelet concentration was used in our study taking into consideration the fact that the platelet concentration is always more than in the white blood cells. It was also noted that the activation of the platelet increases in anti-inflammatory cytokines in the presence of hepatocyte GF.^14^^-^^17^ In order to activate the platelets, calcium chloride was used in the preparation of the PRP for the activation, degranulation and release of growth factors. It was noted that platelet activation plays a major role in exocytosis, cytoplasmic α degranulation with significant burst of growth factor, platelet, derived growth factor, epidermal growth factor, platelet derived growth factor as well as vascular endothelial growth factor.^18^^-^^20^

In our study PRP was used in all etiological groups and it was found to yield remarkably good results as it is well known in the various phases of SSG and plasmatic expiation in the first 24-48 hours, second stage of capillary in growth and the last stage of revascularization taking note of the fact that the grafts viability is critically dependent in the first two phases of the graft take.^21^^-^^23^ The most noteworthy part of the study was that in all the patients treated with PRP, there was instant adherence of the skin graft to the wound bed contrary to the controlled group, where this was absent. The major advantage was the adhesive nature of PRP,^23^ whereas we had to use sutures and staples in the control group. The instant fixation of the skin graft without suturing or stapling resulted in considerable reduction of surgeon’s time required for the removal of sutures and staples at the final stages. In our PRP treated patients, we observed only 10% with graft edema compared to 68% patients in the control group, which had graft edema for more than a week and the most noticeable fact was that the inner dressings as well as the skin graft was observed to be dry in the PRP group. 

The stage of capillary inosculation and early circulation reduced graft edema with application of PRP ^2^^,^^22^ In the PRP group, it was noted that about 1 % of the patients developed hematoma under the graft, requiring secondary grafting as compared to 15% in the control group. In the PRP group, there was a significant reduction in the number of dressings within the first 15 days as compared to the control group which had to undergo frequent dressings within the initial 15 days. This resulted in cost saving in the dressing and the time of the nursing staff and the surgeon and is of considerable advantage in a plastic surgery unit. Our study showed that in the PRP group, scar hypertrophy was not seen mainly due to quick adhesion, less of graft edema as well as the collection under the graft.^23^^-^^29^

The hospital stay of the PRP group was 11 days as compared to the control group which was 17 days resulting in significant cost saving. All the parameters which were studied in our set up were found significantly different between the PRP and the control group. It is considered beneficial in all the aspects with regard to the patient and the surgeon using autologous PRP in grafting, while having considerable practical benefits of effective graft take on the wound beds.

## CONFLICT OF INTEREST

The authors declare no conflict of interest.
